# Material Extrusion of Multi-Polymer Structures Utilizing Design and Shrinkage Behaviors: A Design of Experiment Study

**DOI:** 10.3390/polym15122683

**Published:** 2023-06-14

**Authors:** Abdulsalam Abdulaziz Al-Tamimi, Mehdi Tlija, Mustufa Haider Abidi, Arfat Anis, Abd Elaty E. Abd Elgawad

**Affiliations:** 1 Industrial Engineering Department, College of Engineering, King Saud University, Riyadh 11421, Saudi Arabia; mtlija@ksu.edu.sa (M.T.); mabidi@ksu.edu.sa (M.H.A.); aesayed@ksu.edu.sa (A.E.E.A.E.); 2 Chemical Engineering Department, College of Engineering, King Saud University, Riyadh 11421, Saudi Arabia; aarfat@ksu.edu.sa

**Keywords:** ABS, additive manufacturing, composite structures, material extrusion, multi-material, PLA

## Abstract

Material extrusion (ME) is an additive manufacturing technique capable of producing functional parts, and its use in multi-material fabrication requires further exploration and expansion. The effectiveness of material bonding is one of the main challenges in multi-material fabrication using ME due to its processing capabilities. Various procedures for improving the adherence of multi-material ME parts have been explored, such as the use of adhesives or the post-processing of parts. In this study, different processing conditions and designs were investigated with the aim of optimizing polylactic acid (PLA) and acrylonitrile–butadiene–styrene (ABS) composite parts without the need for pre- or post-processing procedures. The PLA-ABS composite parts were characterized based on their mechanical properties (bonding modulus, compression modulus, and strength), surface roughness (Ra, Rku, Rsk, and Rz), and normalized shrinkage. All process parameters were statistically significant except for the layer composition parameter in terms of Rsk. The results show that it is possible to create a composite structure with good mechanical properties and acceptable surface roughness values without the need for costly post-processing procedures. Furthermore, the normalized shrinkage and the bonding modulus were correlated, indicating the ability to utilize shrinkage in 3D printing to improve material bonding.

## 1. Introduction

The currently available monolithic materials are no longer sufficient to provide the required needs of today’s sophisticated technology and consumer requirements. The demand for custom-made materials to attain superior properties is increasing with advances in technology. Consequently, the development and fabrication of composite materials should be investigated. When two or more constituents are combined at the macroscopic level and are not soluble, the resultant structural substance is called a composite [1]. One of the benefits of composite material is that it is possible to control and tailor its properties to some extent through various combinations and fabrication processes. Composites fall into three different classifications:

- Particle-reinforced (large particle and dispersion strength);
- Fiber-reinforced (continuous and discontinuous);
- Structural (sandwich panels and laminate).

Generally, composites are conventionally manufactured through two processes: open-mold processes (e.g., wet-layup, spray-up, and filament winding) and closed-mold processes (e.g., resin transfer molding, vacuum-assisted resin transfer molding, and injection molding) [2]. However, there are several issues associated with the fabrication of composite materials, such as high labor costs, labor-intensive processes, mold design, and high tooling costs [3]. Additive manufacturing (AM) technology is growing rapidly for the fabrication of composite multi-material structures due to its no-tooling requirements and its ability to fabricate complex shapes [4]. Its advanced multi-material printing capabilities are utilized to fabricate composite structures.

AM is the process of fabricating 3D digital models into 3D physical parts in a layer-by-layer manner. There are seven types of additive manufacturing techniques classified by the ASTM (American Society for Testing and Materials), namely, material extrusion, binder jetting, material jetting, sheet lamination, powder bed fusion, vat photopolymerization, and direct energy deposition [5]. Material extrusion (ME) is one of the most widely utilized techniques due to its affordability, easy setup, user-friendly operation, ability to print complex geometries, recyclability, and minimal material waste [6]. The ME additive manufacturing technique according to the ISO/ASTM 52,900 standard is currently commercially available in three types [7]:

- Fused-filament fabrication (FFF);
- Screw-assisted fused deposition modeling (pellet FDM);
- Direct writing (i.e., bioextruders).

In FFF, successive layers of extruded thermoplastic filament are printed by heating the filament to a semi-liquid condition and extruding it via a micro nozzle according to 3D computer-aided design (CAD) models tessellated in a stereolithography (STL) format [8].

The common materials used in ME are acrylonitrile–butadiene–styrene (ABS), polyethylene (PE), polypropylene (PP), polylactic acid (PLA), polyamide (PA), polycarbonate (PC), polyetheretherketone (PEEK), polyethersulfone (PES), polyphenylene sulfide (PPS), polyetherimide (PEI), polystyrene (PS), polymethyl methacrylate (PMMA), polyethylene terephthalate glycol (PETG), and polycaprolactone (PCL) [9,10]. This technique is particularly focused on eco-friendly polymeric materials with superior physical qualities. Due to the limited mechanical qualities of objects made entirely of thermoplastics, the ME technique has been widely employed to produce conceptual prototypes rather than functioning components. Although slightly enhanced properties can be obtained by optimizing the process parameters, this does not completely eradicate the issue [11].

**Figure 1:**
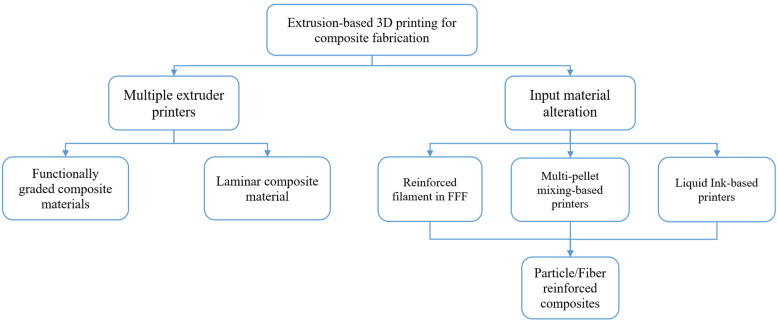
Types of multi-material composite obtained using material extrusion techniques.

Due to their availability, affordability, processability, and generally acceptable mechanical and thermal qualities, PLA and ABS are the two thermoplastic materials most frequently used in ME. ABS excels due to its resistance to strong impact pressures and greater ductility, while PLA has greater material strength and is easier to print [12,13]. However, PLA is brittle, and ABS is relatively difficult to print and is flammable due to its petroleum base, which limits the application of both materials. Inherently, PLA and ABS differ in their printing process parameters, mainly in printing temperature (i.e., the respective nozzle temperature and bed temperature ranges are 190–220 °C and 30–60 °C for PLA compared with 220–250 °C and 100–220 °C for ABS). Parts manufactured from PLA and ABS via additive manufacturing are mostly utilized as working prototypes and concept models. Nonetheless, both are still used in a number of manufacturing tools, consumer items, and spare parts [14,15,16]. These shortcomings would be addressed by combining these materials and creating a PLA-ABS-based composite using multi-material printing technology or multi-material additive manufacturing (MMAM) [12,17]. MMAM is used to enhance the properties of fabricated components and improve the mechanical performance of 3D-printed polymeric-based parts [18]. The composite printing of material extrusion techniques can be classified as shown in Figure 1.

Multi-material 3D printing includes the fabrication of modified filament by reinforcing it with secondary material [19]. However, it is costly to fabricate a multi-material reinforced filament, and high levels of skill and effort are required. Hence, it is important to explore the development of a layer-by-layer multi-material fabrication process that would provide more control in order to alter the final component properties. Additionally, advanced ME 3D printers have the ability to print multiple materials simultaneously via a dual extrusion system [20,21]. This property shows great potential because it enables the integration of different materials with various characteristics—including conductivity, transparency, and chemical resistance into a printed component during a single printing process.

The dual extrusion system is sequentially or nearly simultaneously capable of mixing two or more incompatible materials in the same layer, resulting in a heterogeneous interface with mixed properties [22]. MMAM can be used to print parts as functionally graded materials (FGMs): a form that has the advantages of combined/hybridized material qualities [23]. Additionally, MMAM can be employed by printing a different material in each layer to obtain a laminar composite structure [24]. A study showed that hybrid deposition manufacturing with embedded components can be adapted to fabricate more intricate integrated multi-material components than those fabricated using conventional approaches [25]. New smart 4D structures that can provide precise shapes, qualities, or functions can be printed using MMAM [26]. Rapid manufacturing, customized design, and structural applications will reach a turning point with the advent of multi-material 3D printing. Yadav et al. [27] studied the influences of infill density, material density, and extrusion temperature on the mechanical properties of multi-material parts printed using ME technology. It was reported that infill density and extrusion temperature significantly affected the tensile strengths of the printed parts. Georgopoulou et al. [28] utilized ME to print conductive and non-conductive materials in a sandwich manner to fabricate a soft robotic structure with integrated sensor elements. According to research, fabrication and design parameters can significantly influence how multilateral interfaces interact during 3D printing [27,29]. As a result, these factors should be optimized to obtain enhanced mechanical, thermal, and surface properties. It is also necessary to research the interfacial bonding strengths of multi-material polymer composite parts printed with ME technologies to ensure their structural integrity and mechanical validity. Due to the processing capabilities of ME and the natural behavior of ME materials, material bonding is one of the key problems in multi-material composite printing. Various pre- and post-processes, such as the use of adhesives, interlocking design features, and heat treatment, were taken into consideration to enhance the bonding of multi-material ME parts [24,30,31,32]. In contrast, this paper proposes the building of a multi-material composite structure without requiring pre- or post-processing techniques and with exploration of the effect of polymeric physical behavior (i.e., shrinkage behavior) on the structure’s bonding modulus.

The scope of this paper covers the understanding of the mechanical and shrinkage behaviors of PLA-ABS 3D-printed multi-material composite structures using the FFF material extrusion technique by investigating and optimizing different composite designs (laminate and functionally graded) and printing parameters (layer thickness, printing speed, nozzle temperature, and raster angle). The printing of composite structures was optimized through a design of experiment using the Taguchi method.

## 2. Materials and Methods

### 2.1. Design and Manufacturing

**Figure 2:**
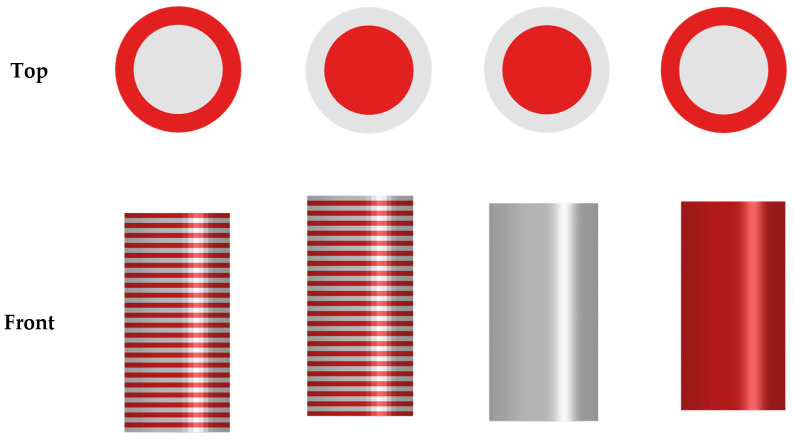

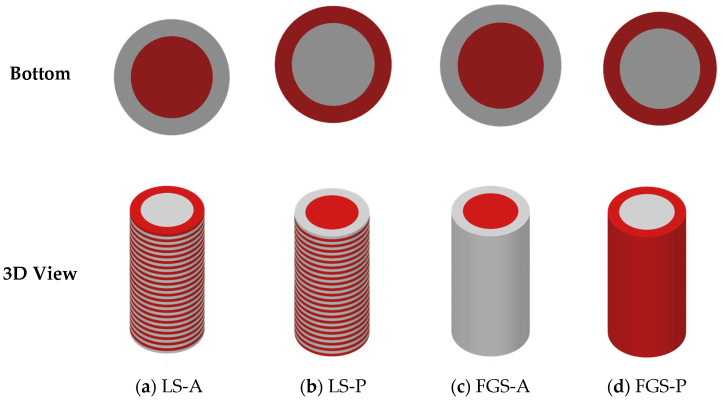
Different views of composite cylinders, showing the laminated sheet designs (**a**,**b**) and the functionally graded structure designs (**c**,**d**). The PLA is shown in red, and the ABS is shown in white.

Two composite designs were proposed and printed for the purpose of investigating the influences of the printing and design parameters of two independent materials in a single structure on its mechanical, surface roughness, and shrinkage properties. The designs were drawn as CAD models according to the ASTM D695-15 standard specimen cylinder shape (12.7∅ × 25.4 mm) and using the Autodesk Inventor (Autodesk, San Francisco, CA, USA) [33]. The first design considers a laminate sheet (LS) structure, shown in Figure 2a,b, composed of two cylindrical sheets that were 0.6 mm thick (the first sheet had ABS as the outer material and PLA as the inner material; the second sheet had PLA as the outer material and ABS as the inner material), and this was repeated until the structure height was achieved. The second design proposed a functionally graded structure (FGS) of two separate cylinders, an outer and an inner cylinder, each consisting of a different material (Figure 2c,d). Both designs were reversed in terms of material allocation to understand the effects of different layering compositions, as shown in Figure 2. The designs were built with an equivalent volume ratio between the PLA and ABS materials.

Polylactic acid (PLA) and acrylonitrile–butadiene–styrene (ABS) (Raise3D Premium PLA and Raise3D Premium ABS; obtained from Raise3D, Irvine, CA, USA) were the materials used to construct the composite structures. An independent dual extruder 3D-printing machine (Raise3D E2; acquired from Raise3D, Irvine, CA, USA) was used to print the composite constructs, with the left extruder head containing the PLA filament and the ABS filament loaded into the right extruder head. The selected printing parameters and their levels in this study are shown in Table 1. The reason for the use of these parameters is to optimize and explore the effect of the shrinkage behavior of the polymers on the bonding of two separate materials. In 3D printing, these parameters are important, as has been reported in several other studies [24,34].

A design of experiment based on the Taguchi approach was proposed to optimize the responses and understand the effects of the printing parameters [35]. The five considered responses—compressive strength, compressive modulus, bonding modulus, surface roughness, and normalized shrinkage—were measured based on the printing parameter variation of an L16 Taguchi orthogonal array, shown in Table 2. After the designs were obtained as CAD models, the files were saved in the stereolithography (STL) format and imported into the Raise3D slicing software ideaMaker 4.3.1 (Irvine, CA, USA) for design pre-processing and the definition of the printing parameters. Two different control specimens, incorporating the four different structures (Figure 2), and a complete solid cylinder were designed and printed in pure 100% PLA and pure 100% ABS using the material manufacturer’s default settings. Common printing parameters were employed for all specimens, such as a 0.4 mm nozzle diameter, vertical orientation, a 100 °C bed temperature, a 100% infill density, and an ABS raft as the build support.

### 2.2. Composite Characterization

#### 2.2.1. Specimen Preparation

Prior to any testing, all specimens were thoroughly cleaned in an ultrasonic bath (Bandelin electronic RK 100 H, Berlin, Germany) filled with distilled water for 15 min at 60 °C before being properly rinsed with 80% ethanol.

#### 2.2.2. Compression Test

A compression test was conducted using an Instron 3369 machine (Instron, Norwood, MA, USA) with a load capacity of 50 kN according to the ASTM D695-15 standard [33]. Tests were carried out at room temperature with a controlled displacement of 1.3 mm/min, and data were recorded at a sampling rate of 50 Hz. A predetermined height of 25% of the original was used as a criterion for stopping the compression tests. This test used five replicates to measure the average compressive modulus and strength. The compressive modulus was represented in megapascals and measured by plotting a tangent of the straight linear portion of the stress–strain curve, then selecting a point of compressive stress and then dividing it by its correspondence strain. Compressive strength was calculated by dividing the compressive load by the specimen at the yield point over the original cross-sectional area and reported in megapascals.

#### 2.2.3. The Bonding Test

**Figure 3:**
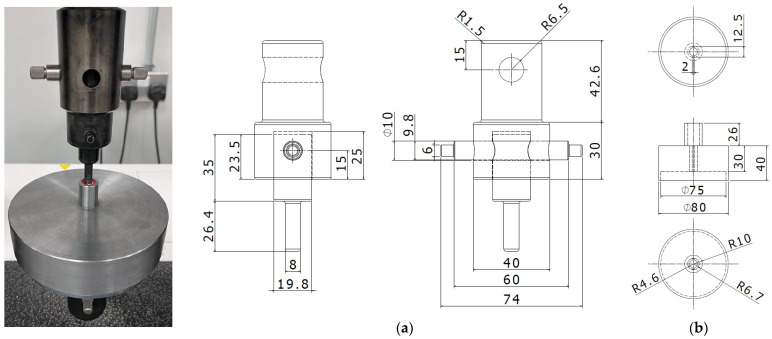
The bonding test apparatus, consisting of (**a**) the pin and (**b**) the cylinder holder.

The bonding modulus between the outer and inner cylinders was employed using a custom-made apparatus (Figure 3) set up in the Instron 3369 machine (Instron, Norwood, MA, USA). This test intended to push the inner cylinder out from the outer cylinder of the specimen. It was performed at the ambient temperature under controlled displacement at a 1 mm/min rate. The predefined conditions for these tests mean that they were conducted until one of the following was met: either the crosshead displacement reached 12.7 mm or the force reached 15,000 N. The resulting forces and crosshead displacement were documented at a sampling rate of 50 Hz for further analysis. The linear portion of the stress–strain curve was calculated as the curve slope to measure the bonding modulus in megapascals. The bonding tests were replicated five times.

#### 2.2.4. Surface Roughness

**Figure 4:**
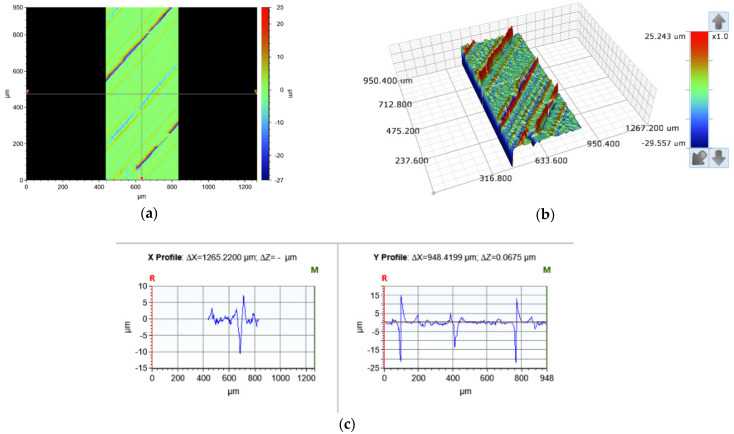
The sample size required to measure surface roughness: (**a**) 2D contour plot, (**b**) 3D contour plot, and (**c**) X and Y direction cross-section plots.

The composite structure surface roughness was measured using an optical profiling system, the Bruker ContourGT-K (Bruker Nano Gmbh, Berlin, Germany), to obtain kurtosis (Rku), skewness (Rsk), Ra, and Rz according to ISO 21920–2:2021 [36]. The size of the sampling area was 400 × 948 µm, as shown in Figure 4. Duplicate measurements were performed for both the top and bottom side of each specimen. The measurements were taken in the interface zone between the PLA and the ABS materials. The surface roughness of the composite structure was measured as follows:

- The arithmetic mean deviation (Ra) was used to measure the arithmetic mean of the absolute ordinate height of the assessed profile (*Z*(*x*)) within a specific sampling length (*l_e_*).
- Kurtosis, Rku, which calculates the mean quartic value quotient of the ordinate height of the assessed profile and the fourth power of the root mean square deviation (*Rq*), was determined using the following equation:

(1) $$Rku=\frac{1}{{Rq}^{4}}\left[\frac{1}{l_{e}}\int_{0}^{l_{e}}Z^{4}(x)dx\right]$$

- Skewness, Rsk, which is the sampling length’s cube of the root mean square and the quotient of the mean cube values of the ordinate height of the assessed profile, was calculated as follows:

(2) $$Rsk=\frac{1}{{Rq}^{3}}\left[\frac{1}{l_{e}}\int_{0}^{l_{e}}Z^{3}(x)dx\right]$$

- Rz measures, along the sampling length, the height summation of the largest peak height and the pit depth of the profile.

#### 2.2.5. Shrinkage Analysis

Normalized shrinkage was calculated assuming dimensional tolerances assigned to diameter and length with an LP modifier and a GN modifier, using a vernier and an optical projector, respectively, according to ISO 14405–1:2016 [37]. Dimensional errors were measured using a two-point method with a Mitutoyo CD-8″ AX digital vernier device (Mitutoyo Corp., Kanagawa, Japan). The absolute values of diameter and length errors were proportional to radial and longitudinal shrinkage, since the nominal dimensions were the same for all specimens. A positive dimensional error corresponds to a positive expansion (negative shrinkage) and vice versa. The vernier measurements were conducted in triplicate. The normalized shrinkage values were calculated using the following equation:

(3) $$Normalized\;shrinkage,\%=\frac{\left(Nominal-Measured\right)}{Nominal}$$

**Figure 5:**
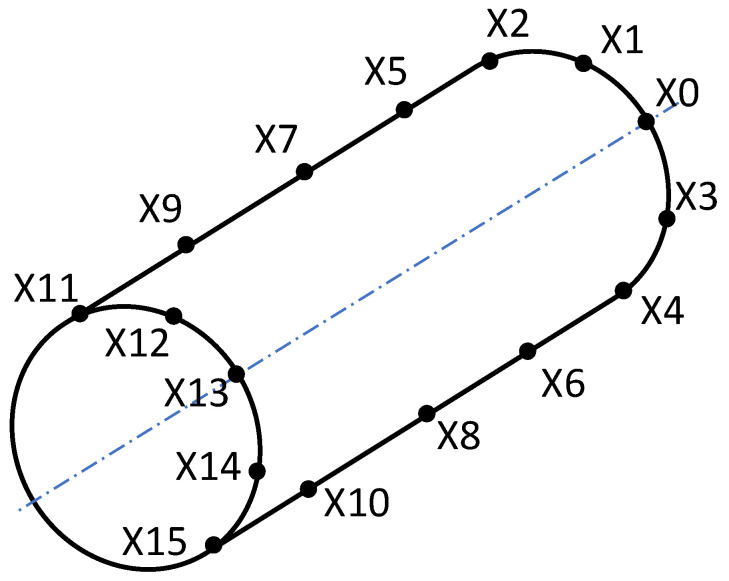
Optical comparator measurement cloud points.

A multi-lens vertical profile projector device, model VOM-2515, obtained from Leader Precision Instrument Co., Ltd. (Dongguan, China), was used to extract sixteen cloud points of specimens, as shown in Figure 5. A mathematically perfect-form feature was fitted to the data point set using the minimum circumscribed association criterion. The oriented bounding box (OBB) tool was associated with the point cloud that represented the bounding envelope containing the actual feature, allowing the length and diameter of the specimen to be determined. The OBB computation algorithm was automated using SolidWorks API and mainly included the following steps: a tessellation was created based on the sixteen cloud points, the barycenters of the resulting triangles were used to determine the covariance matrix, and the OBB orientation was deduced from the eigenvectors of the covariance matrix [38].

#### 2.2.6. Morphological Analysis

The morphology of the composite structure was investigated using a digital microscope equipped with a digital flat camera, model UK1275, and ABSEasyCapture software obtained from Askania Mikroskop Technik Rathenow GmbH (Rathenow, Germany) at a magnification of ×1.25.

#### 2.2.7. Statistical Analysis

Analysis of variance (ANOVA) was conducted on the Taguchi orthogonal array using Minitab 17 (Minitab LLC., State College, PA, USA) to obtain and analyze the statistical behavior of the data. In this analysis, the effect of each factor on each response was considered. The confidence interval was set to 95%.

## 3. Results and Discussion

### 3.1. Shrinkage Behavior

Table 3 depicts the normalized shrinkage percentages for all experiments. The results show that the trends are different for both measurement methods, and this is due to different dimensional specifications. The GN modifier has a different purpose than the two-point measurement (LP) established with the vernier device: the LP interpretation is explicitly local, and the GN application is global [39]. Thus, the resulting shrinkage or expansion is based on the bounding envelope containing the specimen.

In the ANOVA for the vernier measurement, the RA had the highest contribution percentage (60.49%), while the LC contributed 19.31%, as shown in Table 4. The RA significantly contributed to the diameter accuracy of the composite structure, as it controlled the toolpath movement to achieve a circular layer shape. The studied process parameters were effective in terms of the length shrinkage based on the vernier readings. The contributions of the LT, the PS, and the NT were still limited, with low percentages of 8.78%, 3.96%, and 1.86%, respectively, which are similar to the diameter shrinkage. However, the LC became a significant contributor to the length shrinkage (69.88%), while the RA contribution was reduced to 11.59% compared to the diameter shrinkage. According to the machine axis of X and Y (diameter shrinkage), the trend of the layer thickness was proportional to that of the normalized shrinkage. However, in the Z-axis of the machine (length shrinkage), an unexpected trend was found, showing that increasing the layer thickness from 0.1 mm to 0.3 mm would reduce shrinkage and improve printing accuracy. This trend was also observed in other experimental investigations [40,41].

**Figure 6:**
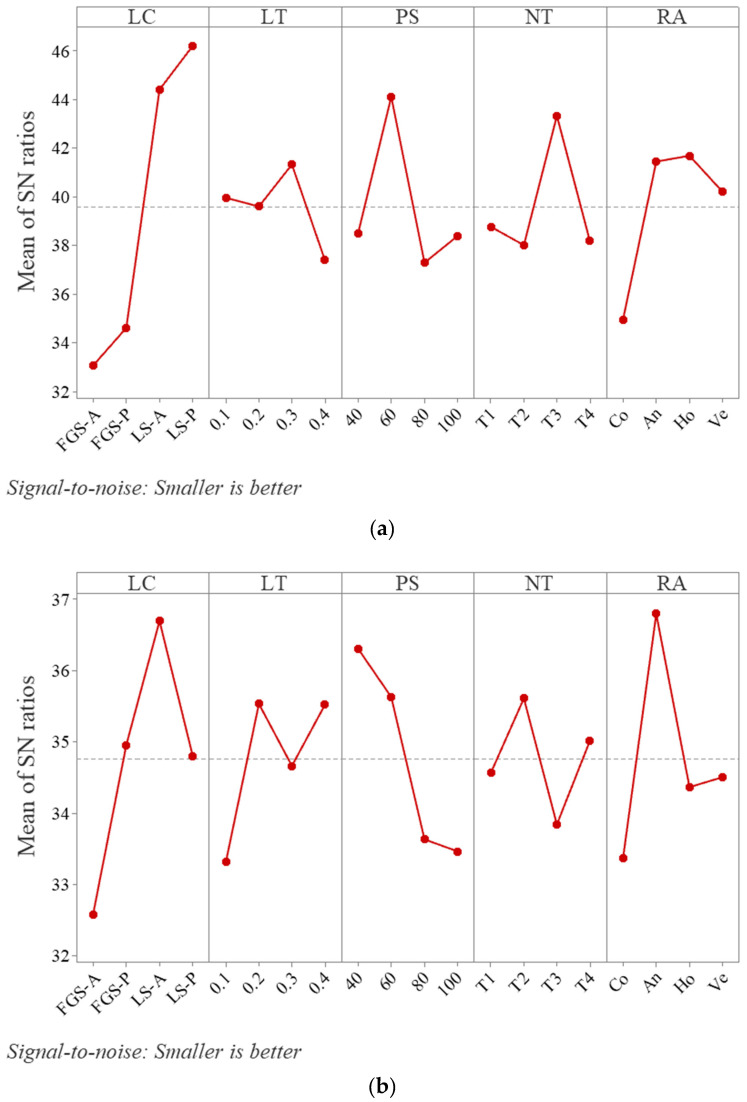
The Taguchi SN ratios for (**a**) vernier and (**b**) projector measurements.

The analysis of the Taguchi design for the S/N outcome of the vernier measurements showed that the LS-P, 0.3 mm, 60 mm/s, 210–255 °C T3, and Ho (90°) as the RA (with no significant difference in the An (45°) raster angle) provided the best parameter combination, minimizing the dimensional errors (diameter and length), as shown in Figure 6. On the other hand, the optical projector for the S/N results showed that the LS-A, 0.2 mm or 0.4 mm, 40 mm/s, 205–250 °C T2, and An as the RA provided the best parameter combination for optimal dimensional accuracy, wherein length and diameter were constrained by global containment.

### 3.2. Surface Roughness Analysis

The surface roughness averages are represented in Table 5. In a normal fashion, the top layer of a 3D-printed part is likely to solidify more rapidly than the bottom one. In fact, the bottom layer was fabricated on a printing support, allowing heat transfer with low cooling, mainly by conduction, with support between adjacent deposing filaments (in addition to convection with captured air and interlayer radiation). The solidification of the top layer is mainly driven by convection and radiation with air [42]. This difference in solidification time yielded a roughness that resulted in differences between the top and bottom sides of the same specimen with the same layer composition in the case of the FGS design. For example, the Rz values of the top and bottom sides of L10 were 5.218 μm and 11.082 μm, respectively. For the LS design, in addition to the difference in heat transfer, the change in the layer structure contributed to the differences in the measurements between the top and bottom layers. Hence, the parameter effects on the surface roughness were analyzed independently.

Rku relates to the tip geometries of valleys and peaks and provides information about further contact between two parts and tribological behavior. The results show that the Rku values are higher than 3, indicating a sharp height distribution. The Rku values of the top surfaces of L2 and L3 are close to 3, implying a normal distribution. In the case of the L14 top surface, the Rku is less than 3, presenting an even height distribution. The above results confirm that the structural support (raft) improved the distribution of the roughness profile to be more rounded, which is similar to results reported by other authors using PLA material, despite the fact that the Rku averages of the top and bottom surfaces were equal to ~4 [34].

Rsk, i.e., skewness, defines the asymmetrical distribution degree in two different categories, with a positive skewness (peak) of >0 representing an empty 3D-printed surface of a material and a negative skewness (valley) of <0 showing the 3D-printed profile of the full material. A normal (Gaussian) distribution is obtained when the Rsk = 0. In most 3D-printing applications, avoidance of positive Rsk is preferred due to the sharp/spiky features that are produced by positive Rsk. As a preliminary analysis, L1, L3, L4, L5, and L10 presented Rsk values of ≤0, while other experiments resulted in Rsk values significantly higher than Rsk > 0.

#### The Effect of Parameters on Surface Roughness

Table 6 shows the contribution of each parameter to the top-layer surface roughness. The process parameters were significantly effective, according to the *p*-values of ≤0.05. A greater layer thickness is expected to produce rougher surfaces due to the increased amount of extruded material [41]. This is why the LT contributed the most to the Ra (contribution percentage of 51.56%), whereas layer composition contributed only 4.10%. Regarding the Rku, the RA generated the highest contribution percentage (34.60%), followed by that of the NT (18.60%). The RA highly impacted the Rsk, with a contribution of 46.30%. In addition, the NT contributed to the Rsk with a value of 22.40%. However, the contribution of the LT to the Rsk was limited to 1.9%. This corroborates previous research where the Rz parameter showed that the LT was considered the most significant influential factor [43]. This conclusion was confirmed in the present study, as the LT contributed 52.70% to the Rz of the top surface, while the PS and LC contributions were limited to 8.80% and 3.70%, respectively.

The contribution of each parameter to the bottom-layer surface roughness outcomes is displayed in Table 7. The ANOVA shows that all considered parameters are statistically significant except for the LC for the Rsk (*p*-value = 0.536). The NT and the LT strongly influenced the Ra values, with contribution percentages of 27.96% and 26.93%, respectively. For the Rku, the contributions of the LC and the LT were relatively low, while the NT contributed 48.80%. The NT and the PS strongly influenced the Rsk, with contribution percentages of 37.64% and 26.92%, respectively. The use of a support beneath the bottom surface reduced the roughness in comparison to the top surface. The LT had a contribution of 29.12% to the Rz, the PS a contribution of 7.97%, the LC a contribution of 15.63%, and the NT a significantly higher contribution of 36.05% due to the required solidification time of the bottom surface compared to the top one, as well as the eventual physical interactions between the ABS support and the bottom layer.

**Figure 7:**
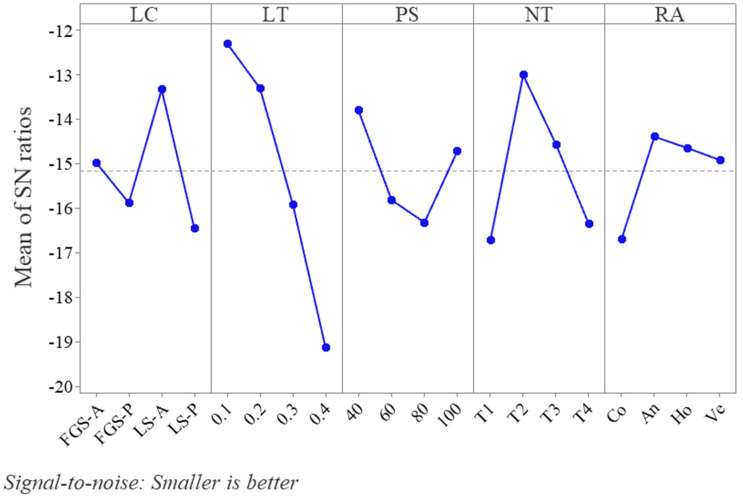
The Taguchi SN ratio for the surface roughness response.

Based on the Taguchi analysis, the signal-to-noise graph of all roughness outcomes (responses for bottom and top surfaces) versus all parameters is presented in Figure 7 and shows that the suitable parameters for lower (better) surface roughness are the LS-A, an LT of 0.1 mm, a PS of 40 mm/min, an NT of T2, and an An (45°) raster angle. The increases in the NT in the PLA and the ABS by only 5 °C (from T1 to T2) significantly improved the surface roughness. Subsequently, the surface roughness gradually deteriorated when the NT varied from T2 to T4. Globally, the surface roughness was inversely proportional to the LT. The concentric raster angle (Co) is clearly not recommended for global surface roughness when compared to the alternatives.

### 3.3. The Effects of Mechanical Properties

#### 3.3.1. Morphological Analysis

**Figure 8:**
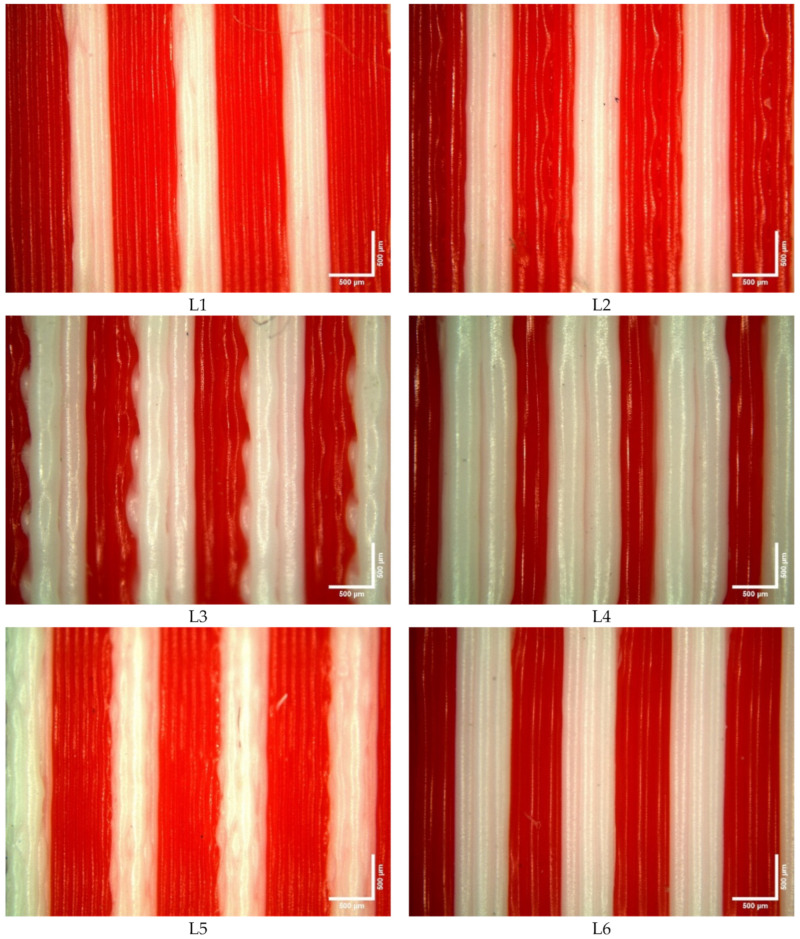

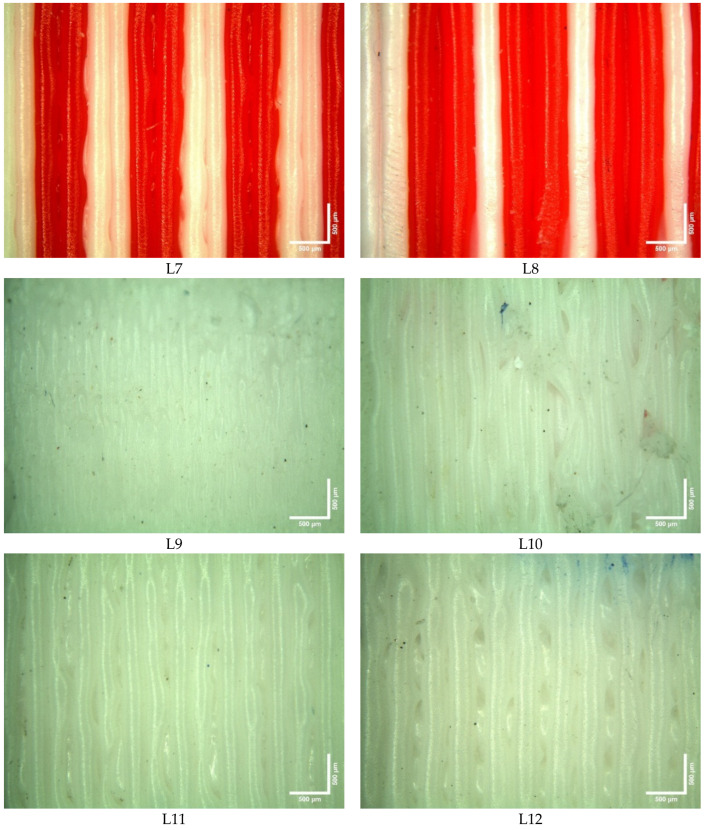

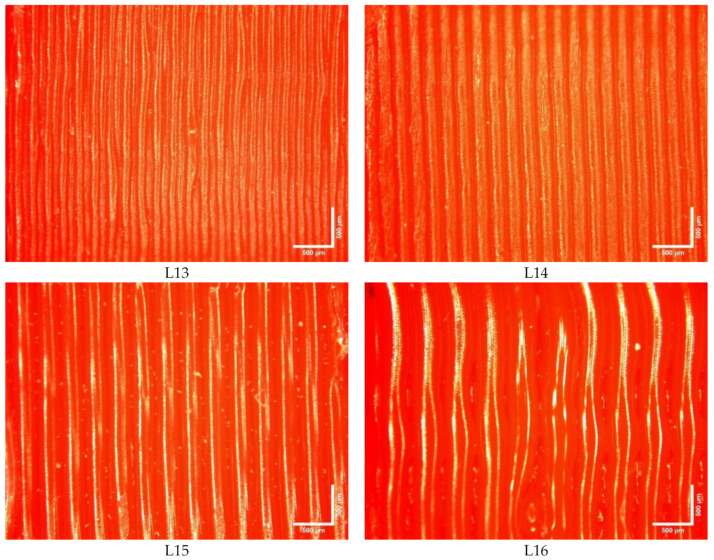
Microscopic images of composite specimens with a scale bar of 500 µm. The PLA is shown in red, and the ABS is shown in white.

**Figure 9:**
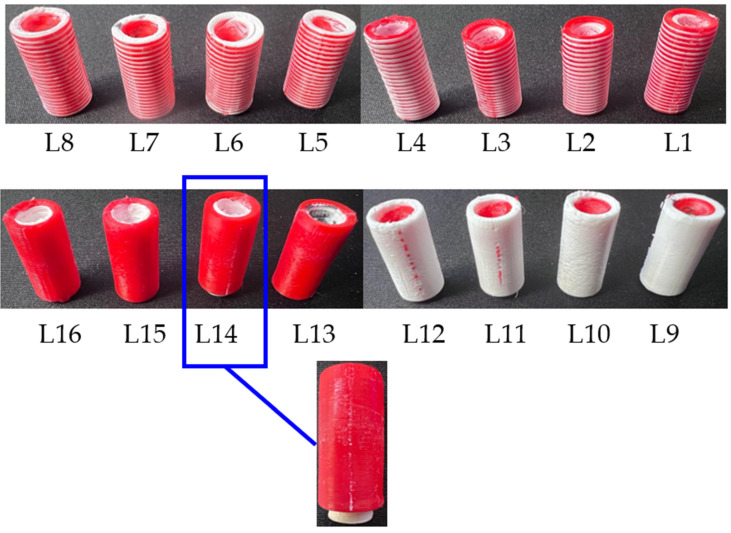
Bonding fracture morphology tests of all sixteen specimens.

**Figure 10:**
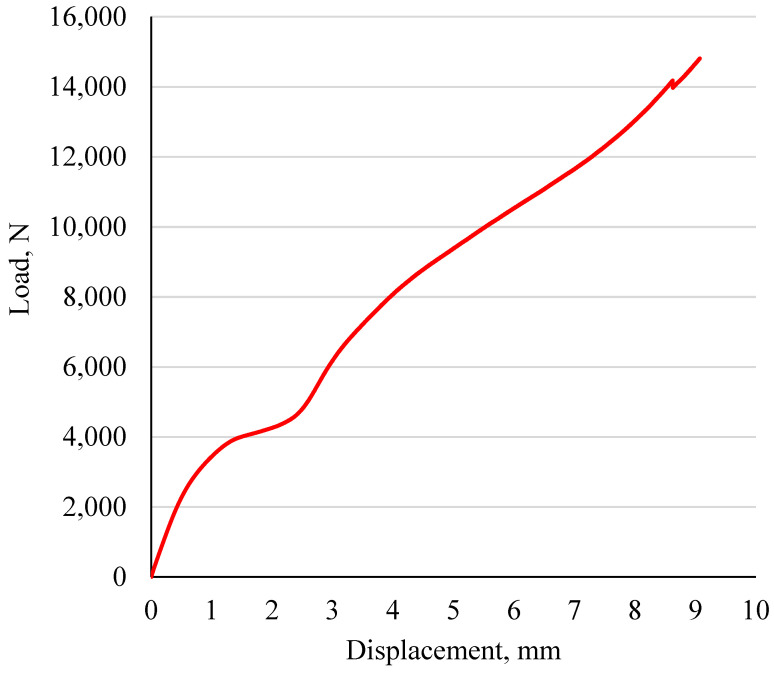
Bonding test load-displacement curve of the L14 specimen.

Microscopic images of the composite structures are shown in Figure 8. Morphologically, all specimens with larger layer thicknesses resulted in higher gaps and disoriented layers, as expected. For example, the L4 specimens showed that the ABS (white) layers were more visible than the PLA (red) layers, and this was the reverse for the L8 specimens. Due to each sheet being set at 0.6 mm and the largest layer thickness being 0.4 mm, the sheet would need to be printed in one and a half steps, and this would cause printing inaccuracy. Disorientation is mostly observed in ABS printing, rather than in that of PLA, due to the PLA material having better printability. The specimen fracture morphology that resulted from the bonding test is illustrated in Figure 9. This test resulted in compressing the inner cylinder region within the structure in all specimens. The findings show that only specimen L14 showed noteworthy separation between the outer and inner cylinders, with an average displacement of ~2 mm. In all other specimens, a non-significant slide was observed between the two cylinders. A comparison of these observations to the average full displacement of ~11 mm showed the custom-made apparatus sufficiency in testing the bonding of the 3D-printed cylindrical composite parts. Additionally, it showed that a composite structure using the proposed designs can withstand high loads without any costly post-processing procedures. A sample of the load-displacement curve of specimen L14 is shown in Figure 10. The results show that the bonding was sufficient to resist a load of 15,000 N in all specimens.

#### 3.3.2. Effect of Process Parameters on Mechanical Properties

**Figure 11:**
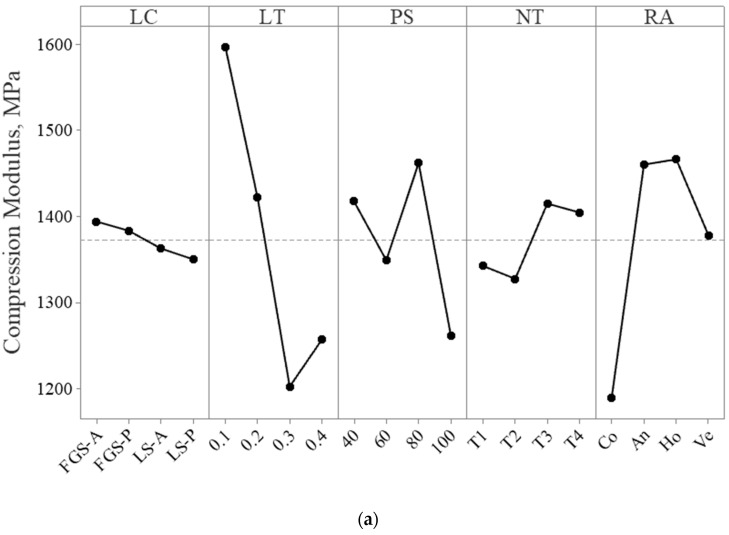

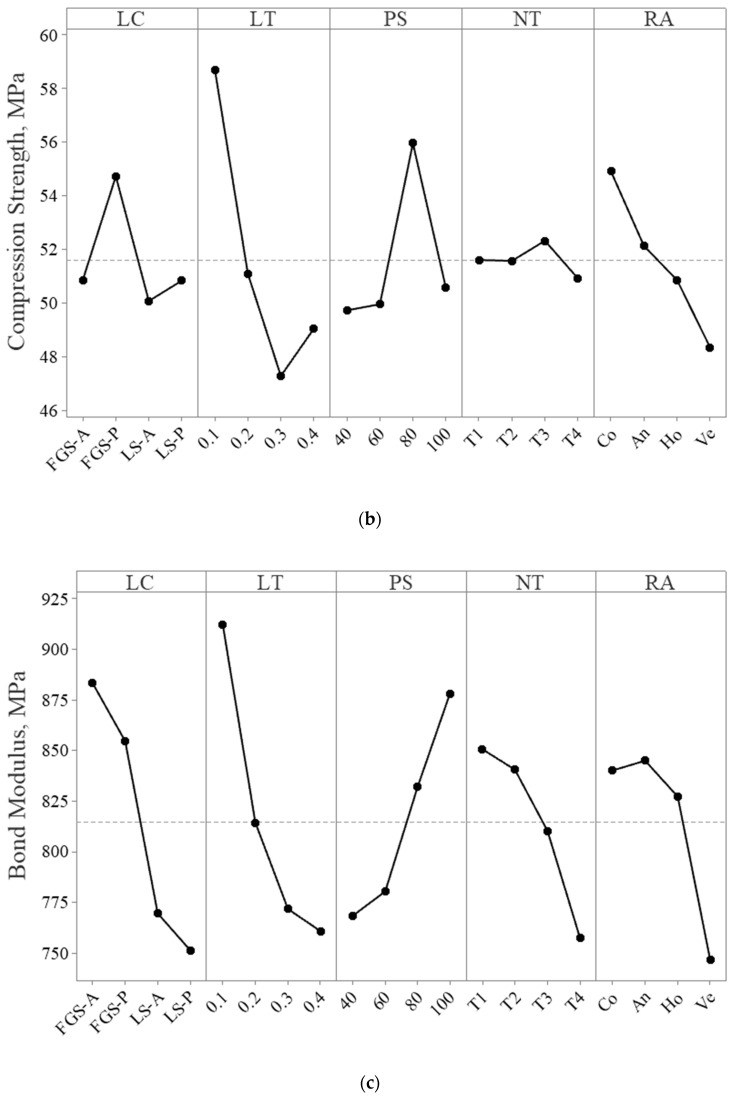
The main effect plots of the (**a**) compression modulus, (**b**) compression strength, and (**c**) bonding modulus.

Table 8 presents the experimental design outcomes for the mechanical properties of the composite structures and the control specimens. The findings of the ANOVA, showing the effect on each factor and its contribution to the mechanical properties, are illustrated in Figure 11 and Table 9. The control specimen results agree with the literature, showing PLA and ABS structures with strengths of 63.4 ± 0.6 and 50.2 ± 0.5 MPa, while their moduli are 2245 ± 17 MPa and 1513 ± 10 MPa, respectively [44]. The control composite specimens in both materials show that slicing the cylinder into different sections and printing it using two extruders would affect its mechanical properties. For example, the LS-P PLA control specimen, in comparison to the control PLA, resulted in a 27% decrease in its compression modulus, while its strength was reduced by 20%. The PLA composite control specimens displayed better mechanical characteristics than the ABS ones. In single-material composite structures, laminate sheet structure printing resulted in better properties compared with the FGS, which was not the case with multi-material printing. The maximum compression strength (62.2 ± 1.5 MPa) and modulus (1822 ± 18 MPa) resulted in experiment L9 (FGM-S, 0.1 mm, 80 mm/s, T4, An) and in experiment L5 (LS-P, 0.1 mm, 60 mm/s, T3, Ho), respectively. The experiment with the most resistance to the bonding test was L13, with 973 ± 31 MPa, and the least resistant structure was L6, with 579 ± 43 MPa. All experiments resulted in lower values than those of the control specimens. However, the composite structures (L1, L5, L8, L9, L10, L13, L14, L15, and L16) showed improved properties, with increases in either their strengths or moduli, in comparison to the ABS control values. Additionally, comparing each control value with the PLA-ABS composite of the same structure, such as the LS-A and L5 or the FGS-A PLA and L9, demonstrates that better mechanical properties can be obtained. This is due to the nature of the composite design, which can be superior to the use of a single material. All factors were significantly effective (*p*-values < 0.05). The results reveal that the LT (52.78%) and the RA (28.14%) affected the compression modulus the most; the LT contributed 52.78% and the PS contributed 18.69% to the compression strength; and the LC and LT contributed 23.94% and 27.57%, respectively, to the bonding modulus.

##### Layer Composition

Layer compositions are mainly split into two categories: functionally graded structures and laminated sheets. Analysis of layer compositions is intended to elucidate the best layering and composing methods in the 3D printing of plastic multi-materials in dual-extrusion FFF ME processes to create composite structures. The levels selected for the categories in this paper were used to investigate the effects on composite structure behavior if printing were initiated using PLA or ABS. The layer composition contributed significantly (*p* < 0.05) in all mechanical tests, contributing a high 23.94% to the bonding modulus outcome but only 0.65% to the compressive modulus and 8.99% to the compressive strength. In all cases, due to the layering process, functionally graded structures produced better mechanical properties and had higher bonding moduli than laminated ones. In the FG structures, the building mechanism layered each cylinder (outer and inner) as one material. On the other hand, the LS structure changed the materials of the outer and inner cylinders after each completed layer. Similar trends for the compression modulus and strength were observed. The results for both the compression modulus and the bonding modulus support the design of FGSs with ABS material, as the outer cylinder (FGS-A) showed improved properties compared to the use of PLA (FGS-P). The maximum bonding modulus resulted in experiments L9 (FGM-A, 0.1 mm, 80 mm/s, T4, and An), with 971 ± 27 MPa, and L13, (FGM-P, 0.1 mm, 80 mm/s, T2, and Ve) with 973 ± 31 MPa. Conversely, the compression strength showed higher values when PLA was used in the design of the outer layer. Overall, PLA performed better in terms of strength; therefore, the use of PLA as an outer cylinder may create a structure that requires larger stresses to yield [45,46].

##### Layer Thickness

Layer thickness was shown to have the highest contribution percentage across all mechanical properties of the composite structures, with ~50% for the compression modulus and strength and 27.54% for the bonding modulus. As expected, a trend of the layer thickness parameter depicting a larger layer thickness would result in lower mechanical properties. The reason behind this is that the layer build-up direction is perpendicular to compression forces, allowing the layers to slide along each other [47]. In addition, a larger layer thickness causes air gaps/bubbles to form between the layers, leading to unwanted porosity in the part depicted in Figure 8 [24]. However, the compression modulus and strength increased again when the layer thickness was set to higher than 0.3 mm. Similar observations were reported in other studies; however, each’s focus was on a single material structure, and different process parameters were set [48,49]. The authors of this work speculate that this was due to a larger air gap, leading to the two materials overlapping during printing.

##### Printing Speed

The printing speed parameter significantly affected the mechanical properties (*p*-values < 0.05). The effect of the PS on the compression modulus and strength varied with every increase in the speed, leading to the highest values when the PS was 80 mm/s. This is logical, as generally, high printing speeds would leave the layer in a semi-molten phase, which would result in better interlayer fusion. On the other hand, higher speeds would provide printing inaccuracy and distortion and would not allow printing beads to develop. Findings have shown that an optimum printing speed of 80 mm/s will produce the maximum compression modulus and strength, while higher printing speeds of 100 mm/s are optimal for bonding between the two cylinders.

##### Nozzle Temperature

The other significant parameter was nozzle temperature, which presented the lowest contribution percentages for the modulus (3.27%), the strength (0.65%) and the bonding modulus (10.20%). Varying the material’s nozzle temperature resulted in local and global maxima of the composite structure modulus and strength. In the literature, these differences are usually linked to polymer fluidity and viscosity and the deposition process of the molten polymer filament [50,51]. The increase in mechanical properties is due to the relaxation of the polymer matrix preventing stretching phenomena and chain orientation. Further, at high nozzle temperatures, the polymer would have higher fluidity and lower viscosity, which would improve the ability of the polymers to fuse with each other. The composite structure bonding modulus would increase while the nozzle temperature decreases, potentially as a result of the different polymer morphology (i.e., the polymeric chain entanglement influence) and strain hardening behavior [30,50]. This means that at higher nozzle temperatures, the composite structure would show a weak bonding modulus due to a loss of strain hardening behavior.

##### Raster Angle

The raster angle parameter contributed a high value (28.14%) to the compression modulus and averaged contributions of 16.72% and 12.35% to the compression strength and the bonding modulus, respectively. Raster angles control the pattern of the nozzle head (toolpath) to deposit the layers. Commonly, it is preferable to design a pattern with a raster angle that is short and along the long axis of the part to improve the mechanical properties [52]. Long and narrow paths would cause the layer printing time to increase, which would consequently create non-uniform printing speeds and uneven nozzle temperatures for the build. This means that the new layer would deposit on a poor previously printed layer, leading to stress accumulation and an increase in porosity or poor interlayer fusion. A higher compression modulus was found in a horizontal “transverse” raster (90°), and higher strength resulted from a concentric raster [53]. In terms of the bonding modulus, an angular (45°) and concentric raster produced the best bonding between the two materials. This is potentially due to the dimensional accuracy and disorientation of the layers that allowed the materials to overlap on top of each other.

#### 3.3.3. Bonding–Shrinkage Effect

**Figure 12:**
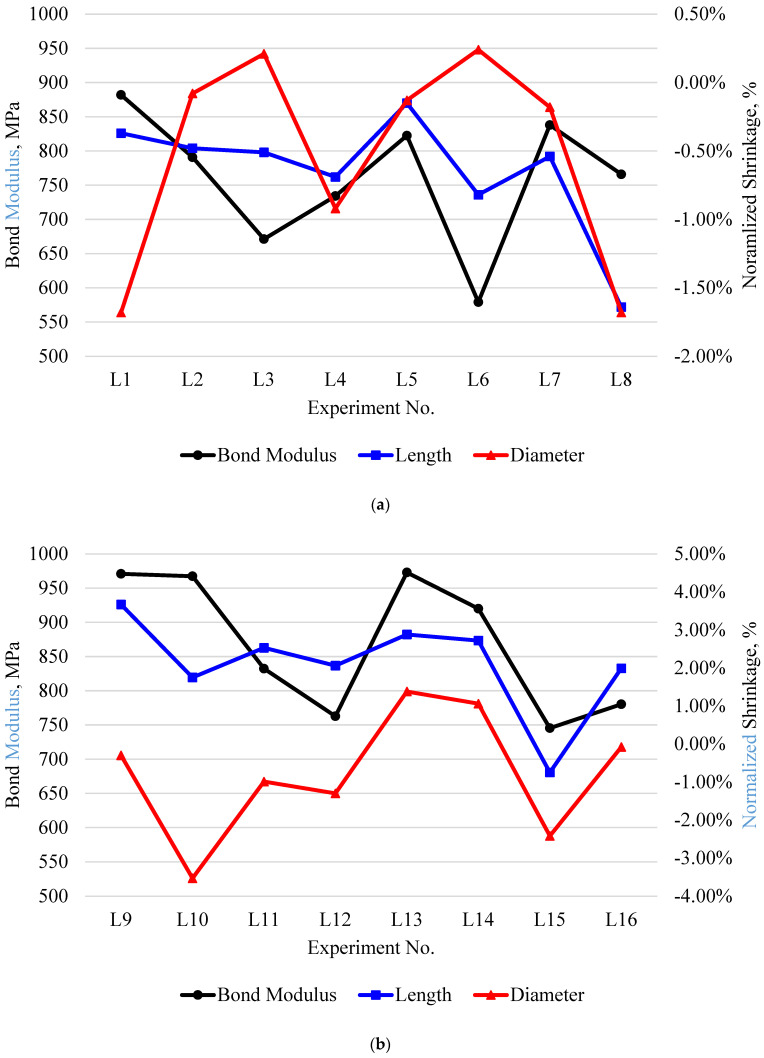
The bonding results for the normalized shrinkages of experiments (**a**) L1–L8 and (**b**) L9–L16.

Figure 12 presents the relationship between the bonding moduli and the normalized shrinkages of the composite structures. The bonding modulus was proportional to the normalized shrinkage along the length of the specimen. This is explained by a loss of dimensional accuracy (high shrinkage), leading to a gap developing within the layer and causing it to collapse more easily. Experiment L11 was the only exception to this trend, as the bonding modulus increased with less normalized shrinkage. On other hand, the diameter-normalized shrinkage presented no apparent trend with the bonding modulus. The laminate structures (L1–L8) were governed more by an inverse relationship of the bonding and normalized shrinkage, while the decrease in the normalized shrinkage of the functionally graded structures (L9–L18) led to a decreasing bonding modulus. The two events could be explained by expanded composite structures, as ≥0% normalized shrinkage results in a larger printed surface area resisting the load to separate the cylinders and vice versa for lower normalized shrinkage. These findings present a direct relationship between normalized shrinkage and bonding, showing that bonding can be improved by controlling the shrinkage of 3D-printed composite parts without requiring any post-processing.

## 4. Conclusions

Currently, composite multi-material 3D printing is recognized as a potential approach that would lead to improved product performance and manufacturing. This paper’s focus is to shed further light on constructing and investigating the 3D printing of polymeric-based composite structures, specifically utilizing part design and shrinkage to improve multi-material printing. Several process parameters were considered (layer composition, layer thickness, printing speed, nozzle temperature, and raster angle) to optimize and understand the printing of composite structures in terms of their mechanical properties, surface roughness, and shrinkage behavior. The optimization of these responses through careful experimental methodology has resulted in the following:

- The normalized shrinkage of composite parts is significantly affected by all considered process parameters, resulting in optimal settings to produce the least dimensional error by employing the LS-P layer composition, a 0.3 mm layer thickness, a 60 mm/s printing speed, a T3 nozzle temperature, and a horizontal (90°) raster angle;
- The best process parameters to produce lower surface roughness are the LS-A layer composition, a 0.1 mm layer thickness, a 40 mm/s printing speed, and nozzle temperatures of 205 °C and 250 °C for PLA and ABS, respectively, as well as an angular (45°) raster angle;
- The optimum configuration for the mechanical properties of the compression modulus and strength and the bonding modulus resulted from experiment L9, which employed a functionally graded composite structure with ABS as the outer layer (FGS-A), a layer thickness of 0.1 mm, a printing speed of 80 mm/s, nozzle temperatures of 215 °C for PLA and 260 °C for ABS, and an angular (45°) raster angle.

Processing 3D-printed multi-material parts is required to prevent material separation in the interaction zone and to improve the material bonding modulus, especially in non-interactive polymers. Parts can be pre-processed through part redesign to include interlocking features that allow the material to mechanically bond. Another means is to add adhesives in the multi-material zone to improve the bonding modulus. On the other hand, post-processing uses heat treatment for the chemical bonding of polymer chains. In contrast, the method in this paper utilized the shrinkage of 3D-printed multi-polymer material to understand the normalized shrinkage and bonding modulus relationship and to improve the bonding in 3D-printed multi-material in order to eliminate the costly pre- or post-processes required. This research has shown that the bonding of two independent materials in 3D printing has a direct relationship with normalized shrinkage, and this can be tailored to eliminate the need for costly pre- and post-processing operations. The composition technique used in this study can be used in printing different shapes and designs using material extrusion techniques to exploit the improved performance of composites over single-material parts. The authors of this work are interested in how this research supports the exploration of 3D printing of multi-material using FFF techniques, and this paper suggests but is not limited to the following possible future research:

- Investigating the possibility of tailoring different composition percentages of materials and using different materials;
- Employing a similar mechanism with a design for additive manufacturing (DfAM) to explore different designs for composite structures and investigating how 3D-printing multi-materials would change the current design of parts and the effect of design on the mechanism;
- Consideration that this paper is purposefully limited to cylindrical shapes, as the authors are interested in additional research on the applications of cylindrical parts, such as improving current PVC water pipes. However, the results of the present study can utilize the advantages of multi-material composites in manufacturing a variety of different shapes of spare parts.

## Figures and Tables

**Table 1:** Printing parameters.

Parameter	Symbol	Unit	Low-Level	Lower Mid-Level	Upper Mid-Level	High-Level
Layer Composition	LC	-	LS-A	LS-P	FGS-A	FGS-P
Layer Thickness	LT	mm	0.1	0.2	0.3	0.4
Printing Speed	PS	mm/s	40	60	80	100
Nozzle Temperature	NT	°C	T1	T2	T3	T4
Raster Angle	RA	°	Co	An	Ve	Ho

LS-A: laminate sheet-ABS; LS-P: laminate sheet-PLA; FGS-A: functionally graded structure-ABS; FGS-P: functionally graded structure-PLA (T1: 200 and 245 °C; T2: 205 and 250 °C; T3: 210 and 255 °C; T4: 215 and 260 °C for PLA and ABS, respectively); Co: concentric; An: angular (45°); Ve: vertical (0°); and Ho: horizontal (90°).

**Table 2:** The Taguchi L16 orthogonal array.

Specimen No.	Layer Composition	Layer Thickness, mm	Printing Speed, mm/s	Nozzle Temperature, °C	Raster Angle, °
L1	LS-A	0.1	40	T1	Co
L2	LS-A	0.2	60	T2	An
L3	LS-A	0.3	80	T3	Ve
L4	LS-A	0.4	100	T4	Ho
L5	LS-P	0.1	60	T3	Ho
L6	LS-P	0.2	40	T4	Ve
L7	LS-P	0.3	100	T1	An
L8	LS-P	0.4	80	T2	Co
L9	FGS-A	0.1	80	T4	An
L10	FGS-A	0.2	100	T3	Co
L11	FGS-A	0.3	40	T2	Ho
L12	FGS-A	0.4	60	T1	Ve
L13	FGS-P	0.1	100	T2	Ve
L14	FGS-P	0.2	80	T1	Ho
L15	FGS-P	0.3	60	T4	Co
L16	FGS-P	0.4	40	T3	An

**Table 3:** Normalized shrinkage in the composite structures.

Specimen No.	Shrinkage, %			
	Vernier		Projector	
	Length	Diameter	Length	Diameter
L1	−0.37%	−1.68%	−0.95%	−2.26%
L2	−0.48%	−0.08%	−0.86%	−0.87%
L3	−0.51%	0.21%	−0.95%	−2.55%
L4	−0.69%	−0.92%	−0.73%	−2.11%
L5	−0.15%	−0.13%	−1.05%	−3.02%
L6	−0.82%	0.24%	−1.09%	−1.64%
L7	−0.54%	−0.18%	−1.06%	−2.20%
L8	−1.64%	−1.68%	−1.95%	−2.09%
L9	3.67%	−0.30%	3.38%	−0.55%
L10	1.75%	−3.53%	1.91%	−4.18%
L11	2.53%	−0.99%	2.55%	−0.76%
L12	2.06%	−1.30%	1.98%	−2.11%
L13	2.88%	1.38%	2.87%	1.49%
L14	2.72%	1.06%	2.80%	0.32%
L15	−0.75%	−2.42%	−0.97%	−2.45%
L16	1.99%	−0.08%	1.66%	−0.39%

**Table 4:** ANOVA for vernier measurements.

Diameter Shrinkage							
Source	DF	Seq SS	Contribution	Adj SS	Adj MS	F-Value	*p*-Value
LC	3	0.001469	19.31%	0.001469	0.00049	39.53	0.000
LT	3	0.000459	6.03%	0.000459	0.000153	12.34	0.000
PS	3	0.000435	5.72%	0.000435	0.000145	11.71	0.000
NT	3	0.000246	3.24%	0.000246	0.000082	6.63	0.001
RA	3	0.004602	60.49%	0.004602	0.001534	123.83	0.000
Error	32	0.000396	5.21%	0.000396	0.000012		
Total	47	0.007608	100.00%				
Length Shrinkage							
Source	DF	Seq SS	Contribution	Adj SS	Adj MS	F-Value	*p*-Value
LC	3	0.009548	69.88%	0.009548	0.003183	189.48	0.000
LT	3	0.0012	8.78%	0.0012	0.0004	23.82	0.000
PS	3	0.000541	3.96%	0.000541	0.00018	10.73	0.000
NT	3	0.000254	1.86%	0.000254	0.000085	5.03	0.006
RA	3	0.001584	11.59%	0.001584	0.000528	31.43	0.000
Error	32	0.000538	3.93%	0.000538	0.000017		
Total	47	0.013664	100.00%				

**Table 5:** Surface roughness responses.

Specimen No.	Top				Bottom			
	Ra, μm	Rku	Rsk	Rz, μm	Ra, μm	Rku	Rsk	Rz, μm
L1	1.448	3.231	−0.127	7.431	1.145	4.131	0.275	6.796
L2	0.617	2.918	0.215	2.990	0.979	3.815	0.244	5.744
L3	0.648	2.969	0.006	3.351	2.385	4.553	−0.244	13.153
L4	3.605	3.355	0.001	18.965	1.345	3.349	0.058	7.360
L5	1.457	3.351	−0.025	7.602	1.427	3.947	0.027	8.697
L6	1.452	4.859	0.216	8.084	1.896	3.852	0.175	9.992
L7	1.239	3.961	0.657	6.330	3.162	4.113	0.052	19.188
L8	5.063	3.854	0.288	27.659	2.194	3.232	0.115	13.144
L9	1.479	3.894	0.239	7.906	1.603	3.699	0.220	9.515
L10	0.943	3.602	−0.133	5.218	1.647	4.005	−0.018	11.082
L11	0.970	4.748	0.458	5.180	1.065	4.092	0.494	7.002
L12	3.599	5.069	0.300	24.229	2.402	3.574	−0.133	18.331
L13	0.613	6.202	0.854	3.453	0.849	3.230	0.333	4.499
L14	1.278	2.304	−0.155	5.205	3.034	3.790	0.131	16.624
L15	4.252	4.624	−0.315	23.185	2.536	3.187	−0.150	14.259
L16	2.191	3.302	0.208	11.379	3.409	5.605	0.119	14.925

**Table 6:** The analysis of variance for top-surface roughness.

Ra							
Source	DF	Seq SS	Contribution	Adj SS	Adj MS	F-Value	*p*-Value
LC	3.000	2.549	4.10%	2.549	0.850	3.710	0.034
LT	3.000	32.474	51.60%	32.474	10.825	47.280	0.000
PS	3.000	4.960	7.90%	4.960	1.653	7.220	0.003
NT	3.000	7.903	12.60%	7.903	2.634	11.510	0.000
RA	3.000	11.430	18.20%	11.430	3.810	16.640	0.000
Error	16.000	3.663	5.80%	3.663	0.229		
Total	31.000	62.978	100.00%				
Rku							
Source	DF	Seq SS	Contribution	Adj SS	Adj MS	F-Value	*p*-Value
LC	3.000	6.449	18.50%	8.175	2.725	12.570	0.000
LT	3.000	2.271	6.50%	4.260	1.420	6.550	0.005
PS	3.000	4.360	12.50%	6.646	2.215	10.210	0.001
NT	3.000	6.490	18.60%	8.619	2.873	13.250	0.000
RA	3.000	12.060	34.60%	12.060	4.020	18.540	0.000
Error	15.000	3.253	9.30%	3.253	0.217		
Total	30.000	34.884	100.00%				
Rsk							
Source	DF	Seq SS	Contribution	Adj SS	Adj MS	F-Value	*p*-Value
LC	3.000	0.448	13.90%	0.264	0.088	8.800	0.002
LT	3.000	0.061	1.90%	0.132	0.044	4.400	0.024
PS	3.000	0.376	11.60%	0.248	0.083	8.270	0.002
NT	3.000	0.723	22.40%	1.195	0.398	39.790	0.000
RA	3.000	1.496	46.30%	1.496	0.499	49.810	0.000
Error	13.000	0.130	4.00%	0.130	0.010		
Total	28.000	3.235	100.00%				
Rz							
Source	DF	Seq SS	Contribution	Adj SS	Adj MS	F-Value	*p*-Value
LC	3.000	74.220	3.70%	74.220	24.741	3.550	0.038
LT	3.000	1054.880	52.70%	1054.880	351.628	50.470	0.000
PS	3.000	175.750	8.80%	175.750	58.584	8.410	0.001
NT	3.000	238.450	11.90%	238.450	79.482	11.410	0.000
RA	3.000	346.750	17.30%	346.750	115.582	16.590	0.000
Error	16.000	111.470	5.60%	111.470	6.967		
Total	31.000	2001.520	100.00%				

**Table 7:** The analysis of variance for bottom-surface roughness.

Ra							
Source	DF	Seq SS	Contribution	Adj SS	Adj MS	F-Value	*p*-Value
LC	3	4.921	22.12%	4.921	1.6402	11.75	0.000
LT	3	5.991	26.93%	5.991	1.9971	14.3	0.000
PS	3	1.462	6.57%	1.462	0.4874	3.49	0.040
NT	3	6.222	27.96%	6.222	2.0739	14.85	0.000
PP	3	1.419	6.38%	1.419	0.4731	3.39	0.044
Error	16	2.234	10.04%	2.234	0.1396		
Total	31	22.249	100.00%				
Rku							
Source	DF	Seq SS	Contribution	Adj SS	Adj MS	F-Value	*p*-Value
LC	3	0.3325	2.93%	0.3325	0.11082	3.74	0.033
LT	3	0.2949	2.59%	0.2949	0.09829	3.32	0.047
PS	3	2.5391	22.34%	2.5391	0.84636	28.56	0.000
NT	3	5.5455	48.80%	5.5455	1.8485	62.37	0.000
PP	3	2.1787	19.17%	2.1787	0.72623	24.5	0.000
Error	16	0.4742	4.17%	0.4742	0.02964		
Total	31	11.3648	100.00%				
Rsk							
Source	DF	Seq SS	Contribution	Adj SS	Adj MS	F-Value	*p*-Value
LC	3	0.01542	1.30%	0.01542	0.005139	0.75	0.536
T	3	0.17091	14.37%	0.17091	0.056971	8.37	0.001
PS	3	0.32019	26.92%	0.32019	0.106729	15.68	0.000
NT	3	0.44771	37.64%	0.44771	0.149236	21.92	0.000
PP	3	0.12615	10.61%	0.12615	0.04205	6.18	0.005
Error	16	0.10892	9.16%	0.10892	0.006807		
Total	31	1.18929	100.00%				
Rz							
Source	DF	Seq SS	Contribution	Adj SS	Adj MS	F-Value	*p*-Value
LC	3	103.7	15.63%	103.7	34.568	10.94	0.000
LT	3	193.19	29.12%	193.19	64.397	20.38	0.000
PS	3	52.86	7.97%	52.86	17.619	5.58	0.008
NT	3	239.14	36.05%	239.14	79.713	25.23	0.000
PP	3	23.9	3.60%	23.9	7.965	2.52	0.095
Error	16	50.56	7.62%	50.56	3.16		
Total	31	663.34	100.00%				

**Table 8:** The average mechanical values of the Taguchi experimental design.

Specimen No.	Compression Strength, MPa	Compression Modulus, MPa	Bonding Modulus, MPa
L1	58.7 ± 0.9	1418 ± 16	882 ± 50
L2	48.5 ± 0.7	1428 ± 20	791 ± 69
L3	47.7 ± 0.3	1328 ± 19	671 ± 27
L4	45.3 ± 0.6	1274 ± 12	734 ± 35
L5	56.3 ± 1	1686 ± 24	822 ± 35
L6	44.8 ± 0.4	1480 ± 18	579 ± 43
L7	46.0 ± 0.3	1125 ± 5	838 ± 16
L8	56.2 ± 0.7	1108 ± 55	766 ± 42
L9	62.2 ± 1.5	1822 ± 18	971 ± 27
L10	53.4 ± 1.5	1189 ± 17	967 ± 28
L11	44.1 ± 0.8	1315 ± 27	832 ±28
L12	42.1 ± 0.5	1212 ± 21	768 ± 30
L13	57.5 ± 0.6	1456 ± 61	973 ± 31
L14	57.7 ± 1	1587 ± 9	920 ± 37
L15	51.3 ± 0.5	1041 ± 79	745 ± 32
L16	51.7 ± 1.3	1464 ± 6	780 ± 46
Control PLA	63.4 ± 0.6	2245 ± 17	/
Control LS-A (PLA)	50.0 ± 0.6	1623 ± 10	820 ± 53
Control LS-P (PLA)	49.8 ± 0.7	1612 ± 24	831 ± 54
Control FGS-A (PLA)	51.5 ± 0.3	1772 ± 5	536 ± 30
Control FGS-P (PLA)	50.6 ± 0.5	1730 ± 14	654 ± 41
Control ABS	50.2 ± 0.5	1513 ± 10	/
Control LS-A (ABS)	39.8 ± 0.5	1081 ± 19	418 ± 36
Control LS-P (ABS)	39.9 ± 0.1	1063 ± 27	400 ± 62
Control FGS-A (ABS)	26.9 ± 0.1	877 ± 5	254 ± 43
Control FGS-P (ABS)	25.8 ± 0.04	847 ± 4	264 ± 3

**Table 9:** ANOVA of mechanical properties.

Compression Modulus							
Source	DF	Seq SS	Contribution	Adj SS	Adj MS	F-Value	*p*-Value
LC	3	22,988	0.65%	18,839	6280	4.59	0.006
LT	3	1,855,483	52.72%	1,826,629	608,876	444.86	0.000
PS	3	450,726	12.81%	465,510	155,170	113.37	0.000
NT	3	115,119	3.27%	123,784	41,261	30.15	0.000
RA	3	990,545	28.14%	990,545	330,182	241.24	0.000
Error	62	84,860	2.41%	84,860	1369		
Compression Strength							
Source	DF	Seq SS	Contribution	Adj SS	Adj MS	F-Value	*p*-Value
LC	3	253.80	8.99%	248.78	82.926	84.15	0.000
LT	3	1489.77	52.78%	1519.78	506.593	514.07	0.000
PS	3	527.67	18.69%	537.33	179.110	181.76	0.000
NT	3	18.30	0.65%	21.29	7.095	7.20	0.000
RA	3	471.96	16.72%	471.96	157.321	159.64	0.000
Error	62	61.10	2.16%	61.10	0.985		
Total	77	2822.61	100.00%				
Bonding Modulus							
Source	DF	Seq SS	Contribution	Adj SS	Adj MS	F-Value	*p*-Value
LC	3	247,176	23.94%	247,176	82,392	45.77	0.000
LT	3	284,672	27.57%	284,672	94,891	52.72	0.000
PS	3	152,788	14.80%	152,788	50,929	28.29	0.000
NT	3	105,367	10.20%	105,367	35,122	19.51	0.000
RA	3	127,490	12.35%	127,490	42,497	23.61	0.000
Error	64	115,200	11.16%	115,200	1800		
Total	79	1,032,693	100.00%				

## Data Availability

No new data were created or analyzed in this study. Data sharing is not applicable to this article.
